# Empowering Caregivers in the Midst of Climate Change

**DOI:** 10.1093/geroni/igab046.2262

**Published:** 2021-12-17

**Authors:** Carole Cox

**Affiliations:** Fordham University, Fordham University, New York, United States

## Abstract

More than 2.7 million children in the United States are raised in kinship families, with the majority of these caregivers, grandparents. Nationally, 1 in 11 children is raised in a kinship family, among Black children, the ratio is 1 in 5. Many of these families struggle economically, welcoming their young relatives into small, often substandard public housing where nonexistent or inadequate heating and cooling exacerbate attempts to moderate extreme temperatures in crowded apartments. For others, responsibility for the children follows the loss of life or permanent disruption of family composition due to weather events such as hurricanes or tornadoes. Grandparent resilience is reflected in their commitment to the well-being of these children, especially observed throughout COVID-19. This presentation will demonstrate that a Grandparent Empowerment Program is an effective strategy to tap into their strength as advocates for adequate health care, educational opportunity, resources, and a world free from climate disruption.

